# Incidence of Arterial Hypertension in People With Periodontitis and Characterization of the Oral and Subgingival Microbiome: A Study Protocol

**DOI:** 10.3389/fcvm.2021.763293

**Published:** 2022-01-07

**Authors:** Mireya Martínez-García, Roberto Carlos Castrejón-Pérez, Adriana Patricia Rodríguez-Hernández, Santiago Sandoval-Motta, Maite Vallejo, Socorro Aída Borges-Yáñez, Enrique Hernández-Lemus

**Affiliations:** ^1^Sociomedical Research Unit, Instituto Nacional de Cardiología Ignacio Chávez, México City, Mexico; ^2^Department of Clinical and Epidemiological Geriatric Research, Instituto Nacional de Geriatría, México City, Mexico; ^3^Laboratory of Molecular Genetics, Graduate Studies and Research Division, School of Dentistry, Universidad Nacional Autónoma de México, México City, Mexico; ^4^Computational Genomics Division, Instituto Nacional de Medicina Genómica, México City, Mexico; ^5^Cátedras CONACYT Consejo Nacional de Ciencia y Tecnología, México City, Mexico; ^6^Center for Complexity Sciences, Universidad Nacional Autónoma de México, México City, Mexico; ^7^Dental Public Health Department, Graduate Studies and Research Division, School of Dentistry, Universidad Nacional Autónoma de México, México City, Mexico

**Keywords:** hypertension, cardiovascular risk factors, chronic inflammation, cardiovascular disease, periodontitis, subgingival microbiome

## Abstract

Cardiovascular diseases are the leading cause of morbidity and mortality worldwide. High blood pressure in particular, continues to increase throughout the global population at an increasingly fast pace. The relationship between arterial hypertension and periodontitis has been recently discussed in the context of its origins and implications. Particularly relevant is the role of the periodontal microbiome linked to persistent local and systemic inflammation, along with other risk factors and social determinants of health. The present protocol will investigate/assess the association between periodontal disease and its microbiome on the onset of hypertension, within a cohort from Mexico City. One thousand two hundred twelve participants will be studied during a 60-month period. Studies will include analysis of periodontal conditions, sampling and sequencing of the salivary and subgingival microbiome, interviews on nutritional and lifestyle habits, social determinants of health, blood pressure and anthropometric measurements. Statistical associations and several classic epidemiology and machine learning approaches will be performed to analyze the data. Implications for the generation of public policy—by early public health interventions or epidemiological surveillance approaches—and for the population empowerment—via the establishment of primary prevention recommendations, highlighting the relationship between oral and cardiovascular health—will be considered. This latter set of interventions will be supported by a carefully planned science communication and health promotion strategy. This study has been registered and approved by the Research and Ethics Committee of the School of Dentistry, Universidad Nacional Autónoma de México (CIE/0308/05/2019) and the National Institute of Genomic Medicine (CEI/2020/12). The umbrella cohort was approved by the Institutional Bioethics Committee of the National Institute of Cardiology-Ignacio Chavez (INC-ICh) under code 13-802.

## 1. Introduction

High blood pressure (HBP) is the leading risk factor for myocardial infarction, cerebrovascular events, atherosclerosis, aortic aneurysms, peripheral arterial disease, heart failure, and chronic kidney disease, among other ailments. Hypertension (high blood pressure) is the most important cause of premature morbidity and mortality and has become a major public health problem (1). Almost 45% of the worldwide population is affected by it and the estimate increases with age in both sexes (2, 3). The etiology of most HBP is multifactorial, resulting from the interaction of renal, neural, cardiac, vascular, and endocrine mechanisms under the influence of genetic, epigenetic, as well as environmental, lifestyle and social factors (4). Additionally, pathophysiological alterations related to microbial dysbiosis, chronic inflammatory states, endothelial dysfunction, increased peripheral vascular resistance, oxidative stress, and immune system responses have been also associated to the incidence and prevalence of hypertension (5–8).

Periodontitis, in turn, is the most common periodontal disease (PD) in adults. Periodontitis has a high prevalence worldwide, it is known to affect around 30–50% of the population in developed countries (9). Periodontitis is a chronic inflammatory disease of infectious origins that has drawn increasing attention as a risk factor to develop hypertension and cardiovascular disease (CVD) (10, 11). Periodontitis is characterized by host-mediated microbe-associated inflammation that results in progressive destruction of the structures that support the teeth (gingiva, cementum, periodontal ligament, and alveolar bone), often leading to tooth loss (3). In particular, the role of the subgingival microbiota in the development of periodontitis, has been clearly identified (12, 13).

Periodontitis and HBP share specific social, economic and even some cardiovascular risk factors as endothelial dysfunction and systemic inflammation (14). However, recent evidence indicates that the association between periodontitis and hypertension may go beyond these factors and may indeed be causal in nature (7, 15, 16). A possible causal relationship has been proposed using Mendelian randomization—a reliable test of causality between risk factors and phenotypes—studied in the UK-Biobank and International Consortium for Blood pressure (ICBP)-Genome Wide Association Study (GWAS) datasets (including 750 000 participants) (16). This study confirmed a link between genetic variants to periodontitis (as approximate lifetime exposure to PD) and BP phenotypes (17).

Nevertheless, the exact mechanisms involved in the association between Periodontitis and HBP remain unknown (17). Clinical and experimental studies have suggested plausible mechanisms related to: local and systemic inflammation, the microbial effect on the vascular system, the host immune response and the specific type of cell activation (18). Certain studies have shown that PD is involved in the pathogenesis of systemic arterial hypertension, identifying the chronic inflammation associated with periodontitis as an independent link with the development and progression of CVD (19–22). Some mediators of chronic inflammation are able to promote subclinical atherosclerosis or endothelial dysfunction with consequences for hypertension. Direct oral microbial effects on the vascular system have also been suggested to affect BP via potential interactions of circulating bacteria with the vascular endothelium (23). Regarding the relationship existing on the triad: imbalance (dysbiosis) of the oral microbiome structure—periodontitis—cardiovascular disease, a number of cross-sectional, case-control, and cohort studies suggest that periodontitis is associated with CVD, specifically with atherosclerosis, by mechanisms involving microbial dysbiosis (24, 25).

To date the strongest hypotheses about the pathogenic route underlying this association suggest that the relationship is given by a progression of microbial dysbiosis. This, in turn, favors the development of periodontitis by generating chronic inflammation, disrupting the immune response (a phenomenon called immune subversion) and increasing the atherogenic potential (11, 18). Emerging experimental animal evidence indicates that immune system activation induced by a keystone periodontal pathogen (*Porphyromonas gingivalis*) promotes the development of hypertension, vascular inflammation, and endothelial dysfunction (3).

Despite this association between periodontitis and high blood pressure (26, 27), few longitudinal studies have been conducted to date, often with inconsistent results, as reported by Surma et al. (28). Some researchers have observed a temporal association between periodontitis and the incidence of HBP (6), but others have found no association between periodontitis and any risk conditions for cardiovascular disease (29–31). However, intensive treatment of severe periodontitis improves endothelial function by reducing systemic inflammation (decrease of proinflammatory markers and cytokines) in patients with and without other comorbidities (3, 23, 32).

The actual association between periodontal disease and hypertension is not new and several studies beyond the purely observational (such as the already discussed Mendelian randomization (16)) have ascertained it. However, one novel aspect of our study is that, in addition to analyze this association, we are interested in studying the role that the periodontal microbiome may be playing in both, initial and sustained infection; as well as in the modulation of the immune and inflammatory responses that lead to the systemic effects that are known (or hypothesized) to be behind the association between periodontal disease and hypertension.

After further confirmation via long-term longitudinal studies, periodontitis may represent a novel modifiable risk factor for hypertension (3). However, there are still profound gaps in our knowledge of what are the functional aspects that lead to dysbiosis in the oral subgingival microbiome, its relationship with periodontitis and other pathologies that have been associated with cardiovascular disease and hypertension (33). Longitudinal cohort studies are required to assess periodontitis exposure, associated risk factors, and the incidence of HBP. Along these lines, the purpose of this study is to contextualize the association between hypertension and periodontal disease, in particular by considering the role that microbial dysbiosis may have on each of these conditions, and on their interplay.

## 2. Scope of the Project

### 2.1. Main Goal of the Study

The main objectives of this study are: (i) to identify the subgingival microbiome, as well as the factors associated with periodontitis, (ii) to quantify its association with the incidence of hypertension and (iii) to analyze the triple association between the oral subgingival microbiome, the presence and extent of periodontal disease, and the development of hypertension in people aged 20 years and over that will be followed for a period of 60 months.

The primary endpoint is to determine the association of the subgingival microbiome profiles with the presence of hypertension and periodontitis. Secondary goals include the assessment of the relationship between periodontitis and hypertension in the socio-demographic context of the metropolitan population of Mexico City, as well as the characterization of the subgingival microbiome in periodontal health and periodontitis conditions of such study sample.

### 2.2. Specific Goals

Specifically we will reach the above mentioned goals through the following steps:

1. Estimating the prevalence of periodontitis in people aged 20 years and older at baseline in the cohort under study.
2. Identifying differences in the distribution of known cardiovascular risk factors (age, gender, weight, physical activity, smoking habit, and stress) and social determinants of health (education, occupation, and marital status), in individuals with and without periodontitis at the beginning of the study.
3. Estimating and assessing the impact on the quality of life of individuals with varying degrees of periodontitis at the beginning of the study.
4. Identifying differences in the perceived need for dental treatment and the use of dental services between individuals with and without periodontitis at the beginning of the study.
5. Studying the association between the subgingival microbiome profiles and the periodontal health conditions or periodontitis at baseline.
6. Identifying whether cardiovascular risk factors and social determinants of health are associated with the risk for periodontitis and with the 60-month incidence of arterial hypertension in participants of the study.
7. Analyzing the distribution of xerostomia, the use and functionality of removable prostheses, the presence of root remains, the number of present natural teeth, the number of teeth with caries, as well as the oral hygiene habits in persons with and without periodontitis.
8. Identifying if there is any association between the 60-month incidence of hypertension and the baseline salivary and subgingival microbiome and the baseline presence of periodontitis.
9. Analyzing the relative abundance of microbial species profiles in the salivary and subgingival microbiome and its relationship with arterial hypertension within a 60-month follow-up.
10. Contrasting the epidemiological evidence of the relationship between periodontitis and hypertension with the findings of the analysis of the subgingival periodontal microbiome.

### 2.3. Hypotheses

The main hypotheses motivating the design of this project may be summarized as follows:

1. Individuals with periodontitis have an augmented risk of developing arterial hypertension than people without periodontitis, within a 60-month period controlling for: sex, age, smoking, and oral hygiene habits; as well as social determinants of health such as education, occupation, and marital status.
2. The relative abundance of microbial species profiles in the subgingival periodontal microbiota of participants with and without periodontitis will be different.
3. The relative abundance of microbial species profiles in the salivary and subgingival periodontal microbiota of participants that develop arterial hypertension will be different to that of the ones not developing HBP.

## 3. Methods

### 3.1. Ethical Aspects

In accordance with the Regulation of the General Law of Health in Matters of Health Research, in the Second Title (Of the Ethical Aspects of Research in Human Beings) article 17, this research is classified among the Investigations with Minimum Risk, since no adverse effects have been reported from periodontal measurement on teeth (34). Written informed consent will be required. The confidentiality of the information will be ensured and each of the participants will be given a health diagnosis.

We must highlight that all participants will be given detailed information and recommendations about their individual health (blood pressure levels, lab tests and periodontal evaluation) immediately after every evaluation and referred to specialized treatment whenever needed. However, we should highlight that receiving medical and/or periodontal treatment will depend only on each participants' decision.

### 3.2. Study Population

**Figure 1:**
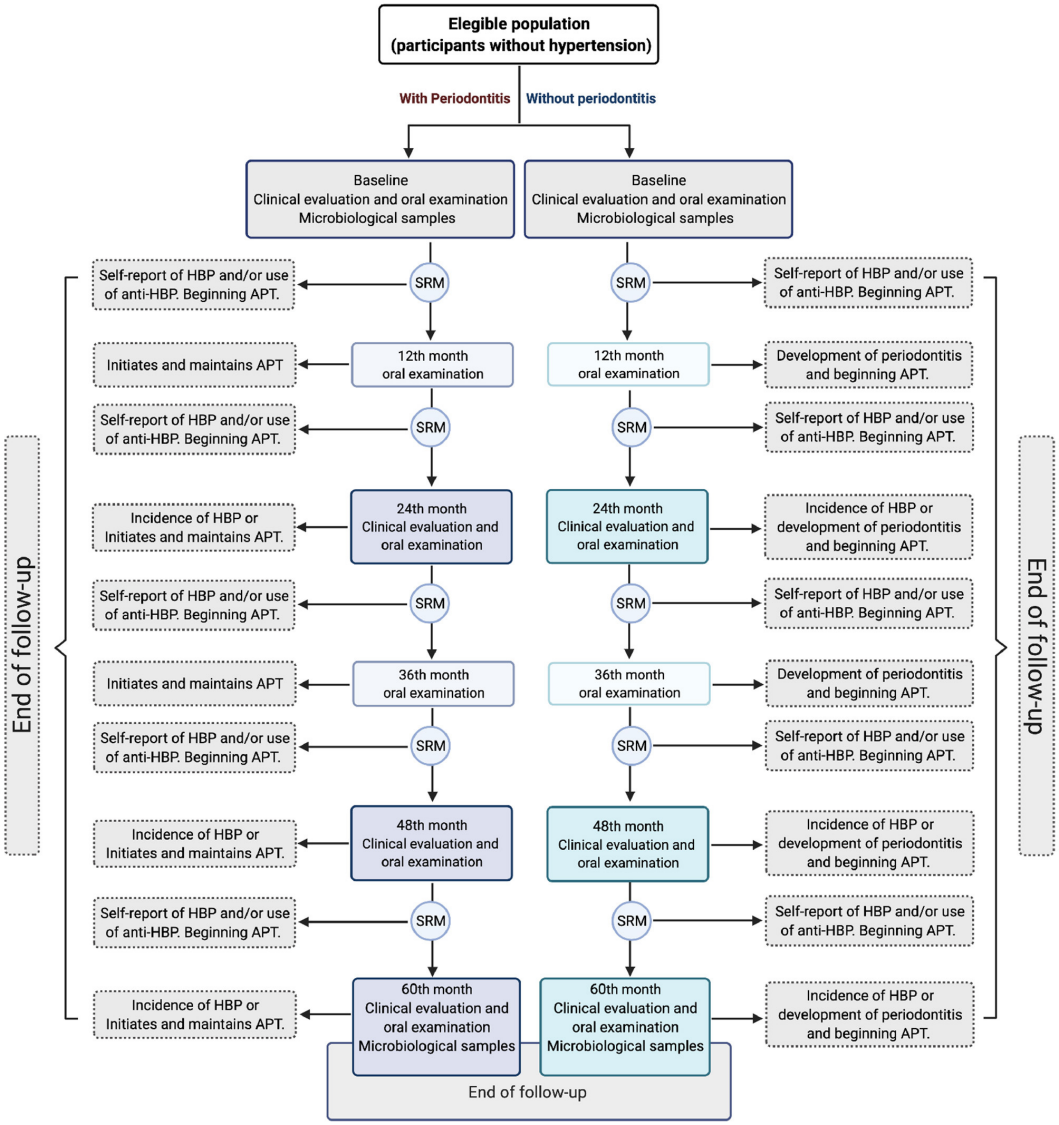
Outline of the study 60-month follow-up. Different scenarios are presented according to the participant role. *HBP*, High blood pressure; *SRM*, Semiannual remote monitoring; *APT*, Active periodontal treatment. The image generated by the authors was created with the *BioRender* App.

This will be a cohort design, with the presence of periodontitis as an exposure factor for the development of hypertension (see Figure 1). This protocol was submitted and approved by the Ethics and Research Committee of the School of Dentistry, National Autonomous University of Mexico (SD-UNAM), the approval key is CIE/0308/05/2019 and the National Institute of Genomic Medicine (CEI/2020/12). The umbrella cohort was approved by the Institutional Bioethics Committee of the National Institute of Cardiology-Ignacio Chávez under code 13-802.

#### 3.2.1. Participants and Recruitment

The study population is a sub-cohort of the participants in a cohort from the Mexico City metropolitan area (35). It is a prospective longitudinal study in which clinically healthy residents of Mexico City, between 20 and 50 years old are recruited to be evaluated every 2 years for a period of 10 years or until they develop hypertension. Anthropometric (weight, height and waist circumference), clinical and biochemical parameters (blood and urine laboratory tests with markers such as glucose, cholesterol, triglycerides, sodium and potassium, etc.), nutritional and lifestyle habits (physical activity, sleep quality, alcohol and tobacco consumption) and social determinants of health are assessed (Supplementary Material). All participants are measured periodically (every 2 years) by a battery of specific serum biomarkers of systemic inflammation. Participants will be contacted every 6 months via email or by telephone to assess their medical and dental history including treatment for hypertension and/or periodontitis, as well as any medication (including antibiotic treatment) used over the previous months (see Figure 1).

### 3.3. Sample Size

#### 3.3.1. Sample Size for Periodontitis and Incidence of Hypertension

The projected sample size for periodontitis and incidence of hypertension will be 1212 individuals from those participating in the umbrella cohort. A logistic regression (LR) for a binary response (hypertension) on an independent variable (periodontitis) with a sample size of 866 participants (90% of whom are in group X = 0 and 10% are in group X = 1) achieves 90% power with 0.05 significance to detect a change in the Probability (Y = 1) of an estimated value of incidence of hypertension of 21.5%. This change corresponds to a Relative Risk (RR) of 2.4 (a RR between 1 and 2.74 has been reported) (6, 30, 36). An adjustment was made since the multiple regression of the variable of interest in the other independent variables in the LR obtained a *R*^2^ of 0.3. A 40% dropout rate is expected (*n* = 346), so the projected sample size will be 1,212 people.

#### 3.3.2. Sample Size for the Microbiological Studies

The sample size of the oral and subgingival microbiome study is projected to be 200 people sub-sampled in a randomized adjusted cohort design (100 subjects with periodontitis and 100 without periodontitis). This is so since the lower bound for the number of samples to probabilistically infer co-existence networks (in this case of microbial species) by the method we use (based on the calculation of the mutual information function) is ~100 per evaluated group (37). A total of 400 microbial samples per evaluation time (baseline and 60 follow-up months) will be placed 200 for periodontal health subjects and 200 for periodontitis subjects, with a subdivision of 100 for saliva and 100 subgingival samples each. Since the sequencing analysis for microbiota is relatively expensive it was decided to use just this number for experimental design. Sampling for microbiome analysis will be done in participants who have developed hypertension (with periodontitis and without periodontitis) and in those who have remained at normal blood pressure levels (with periodontitis and without periodontitis).

### 3.4. Selection Criteria

To pre-select participants candidates to sampling, a standardized questionnaire regarding general health will be applied, including the presence of concomitant systemic diseases, daily intake of prescribed medication, as well as a *Self-reported assessment of Periodontitis*, self-perception of gingivitis, dental care, and oral hygiene habits (38).

#### 3.4.1. Inclusion Criteria

- Women and men between 20 and 50 years old.
- People who live in Mexico City.
- People without hypertension.
- People who do not take medications that affect blood pressure.
- People without chronic infections, inflammatory, immune disorders, or other systemic conditions such as diabetes.
- People who agree to participate in the study and sign the informed consent.

#### 3.4.2. Exclusion Criteria

- People diagnosed with cancer with an effect on survival.
- Pregnancy or lactation.
- People with cognitive and mental disabilities.
- Edentulous people.
- People with dental implants.
- People who are undergoing systematic and regular periodontal treatment.
- People with forms of refractory or progressive periodontal rupture, periodontitis in a rapid rate of progression, necrotizing periodontal diseases or periodontal abscess that require regular taking of antibiotics.

#### 3.4.3. Elimination Criteria

- People who develop hypertension and initiate and/or maintain regular and active antihypertensive therapy.
- People who develop systemic and uncontrolled diseases that may interfere with the severity of periodontitis.
- People who initiate and maintain regular and active periodontal treatment during the study follow-up.
- People who develop some forms of periodontitis that require regular antibiotics.
- People who undergo dental procedures that involve implant placement.

### 3.5. Variables (Identification, Definition, and Measurement Scales)

#### 3.5.1. Definition of Variables

- Dependent-variable of interest: Incidence of hypertension.
- Independent-variables of interest: Periodontal-related parameters and oral-related microbial profiles.
- Oral health covariates: Oral health related quality of life; diet changes; perception of need for dental treatment; xerostomia; use and functionality of removable dental prostheses; oral hygiene (presence of biofilm and dental calculus); number of natural teeth present; dental caries; root debris and gingival bleeding.

### 3.6. Dependent Variable

#### 3.6.1. Systemic Arterial Hypertension

Blood pressure assessment will be performed every 2 years in accordance with the *2020 International Society of Hypertension Global Hypertension Practice Guidelines* (39). Hypertension will be diagnosed when (1) a person's systolic blood pressure (SBP) in the office is ≥140 mm Hg and/or their diastolic blood pressure (DBP) is ≥90 mm Hg after at least 3 repeated examinations and (2) when a person's systolic blood pressure (SBP) at home (HBPM) is ≥135 mm Hg and/or their diastolic blood pressure (DBP) is ≥85 mm Hg. Classification values are charted below (39).

##### 3.6.1.1. Office Blood Pressure Measurement Classification (SBP/DBP, mm Hg)

- Normal BP, <130 and <85
- High-normal BP, 130–139 and/or 85–89
- Grade 1 hypertension, 140–159 and/or 90–99
- Grade 2 hypertension, ≥160 and/or ≥100

*3.6.1.1.1. Preparation and Position*. Before office BP measurements, participants will be advised to have an empty bladder, not to exercise and not to smoke, drink coffee or tea, for at least 30 min on the visit day. Participants will be seated in a quiet room (neither participant nor staff should talk before, during and between measurements) with comfortable temperature for at least 5 min before the first evaluation, arm resting on table with mid-arm at heart level; back supported on a chair; legs uncrossed and feet flat on the floor (39).

*3.6.1.1.2. Monitoring Schedule*. BP will be measured in the right arm three times with 1 min between them with a validated electronic (oscillometric) upper-arm cuff device (40–42) according to the individual's arm circumference. The average of the last 2 measurements will be calculated. If one of the three measurements is remarkably different, a fourth measurement will be taken. If BP of first reading (first three measurements) is less than 130/85 mm Hg no further measurement is required (39).

##### 3.6.1.2. Blood Pressure Measurement Plan According to Office Blood Pressure Levels

Repeated examination and/or measuring BP levels at home will be performed as follows:

- Participants with normal BP (<130/ <85), will be re-examined on at least one further office visit (1–2 week intervals).
- Participants with suspected high-normal BP (130–139 and 85–89), will have repeated office visits (2 at 1–4 week intervals) required and will be asked to monitor blood pressure for at least 5 (preferably 8) consecutive days at home (HBPM).
- Participants with suspected hypertension (≥140 and ≥90), repeated office visits (3 or 4 visits at 1–4 week intervals) for re-examination will be required. Also, they will be asked to monitor blood pressure for 8 consecutive days at home (HBPM). Those diagnosed with HBP during this period (see HBPM below) will be referred to the ambulatory blood pressure monitoring (ABPM) service at the INC-ICh.
- The diagnosis will be made on a single visit, if BP is ≥180/≥110 mm Hg on the first set of three measurements. Then the participant will be immediately scheduled for a clinic visit at the INC-ICh to receive appropriate treatment. The day of the diagnosis of hypertension will be considered the date of disease onset.

Participants who are diagnosed with HBP at another facility at any time during the study will be requested to immediately notify principal investigators. These participants will be asked to schedule a visit at the INC-ICh prior to starting any medical treatment, to confirm the diagnosis and to complete the “end of the study” visit. Also, participants who are diagnosed with HBP and initiate and maintain regular antihypertensive therapy (more than 2 weeks) will be eliminated, according to the criteria of the umbrella cohort (35). The date of disease onset will be considered as the date of confirmation of the diagnosis and/or the date of initiating antihypertensive therapy.

For those diagnosed with HBP during one of the follow-up visits at INC-ICh, the date of disease onset will be recorded as the date of the visit. Additionally, all participants will be contacted by telephone or by email every 6 months to verify whether they have been diagnosed with HBP and/or if they are taking antihypertensive therapy (see Figure 1).

##### 3.6.1.3. Home Blood Pressure Monitoring

In order to reduce the number of possible false readings (high possibility of white coat or masked hypertension), high-normal BP and hypertension cases will be instructed, trained and asked to monitor blood pressure at home. The participants will be guided on how to select an appropriate HBPM device, how to measure their own BP (preparation, position and monitoring schedule) and will be provided some practical recommendations for optimal HBPM performance (43–45).

*3.6.1.3.1. Preparation and Position*. The participant should avoid smoking, caffeine and exercise for 30 min before measurements. They should have an empty bladder, and rest quietly in a comfortable seated position with the back supported, legs uncrossed, and both feet on the floor for 5 min before taking home BP measurements. The cuff of the device should be placed directly above the elbow and pulled taut, with the arm supported on a flat surface. Avoidance of talking or using electronic devices during BP measurements will be required. The participant will be instructed not to share the device with anyone unless this is equipped to store readings for multiple people and carry out a periodic check-up of the device.

*3.6.1.3.2. Monitoring Schedule*. HBPM will be based on 2 measurements taken at least 1 min apart in the morning and evening. Morning measurements should be made before breakfast and evening measurements should be taken before going to bed.

Current guidelines recommend that the average of all these values should be used with the exception of the first day, which should be discarded for clinical decision-making. If there is a discrepancy between office BP and home BP readings, a home BP-based diagnosis should take precedence (46). HBPM reference values are as follows:

##### 3.6.1.4. Home Blood Pressure Measurement (SBP/DBP, mm Hg)

- Hypertension, ≥135 and/or ≥85 (participant will be sent to clinical visit and ABPM for follow up)

#### 3.6.2. Periodontitis

The periodontal evaluation will be done every year in terms of probing pocket depth (PPD) and clinical attachment loss (CAL). Examining 6-site-per-tooth in all natural teeth—except third molars–, measuring the distance from the cemento enamel junction (CEJ) to pocket depth and the distance from the gingival margin to the CEJ will be performed. Participants will be divided into two groups: (1) participants without periodontitis and (2) those with a diagnosis of periodontitis (mild, moderate and severe) based on *Centers for Disease Control and Prevention and American Academy of Periodontology* (CDC/AAP) definition (47), suggested for epidemiological studies by Holtfreter et al. (48). According to a recent agreement among experts from Europe and USA (48) and recent epidemiologic trends, the 2012 CDC/AAP criteria become a consensus approach for epidemiological studies worldwide (49). Furthermore, the 2017 World Workshop on the Classification of Periodontal and Peri-Implant Diseases and Conditions (50), in Appendix B, recommended that *After a long-standing debate on how to provide data to inform surveillance of periodontal disease, a recent multinational effort has suggested standard reporting of periodontal epidemiology surveys. Such efforts are important to provide comparable data across populations*.

Several international research efforts have actually made use of this definition for periodontitis cases. For instance, a recent study (51) that compared CDC/AAP Case Definition and the EFP/AAP Classification concluded that use of the EFP/AAP classification (as a possible new globally accepted classification) to assess periodontitis prevalence, severity and risk factors when conducting epidemiological surveys may not be totally reliable for use in population surveys. Hence the use of CDC/AAP in these studies is deemed more appropriate. Also, a recent systematic review and meta-analysis of the association of periodontitis with hypertension determined the CDC/AAP as a confident case definition of periodontitis (3).

The implementation of this classification improved reporting quality and permitted meaningful comparisons of the prevalence of periodontal diseases across populations in epidemiological studies (48). Indeed, many recent epidemiological studies with diverse populations in different parts of the world have used this classification (52–57), as well as (58–61) and (61–63).

For the sake of the present research and based on the international consensus just discussed, periodontitis cases will be classified as follows:

- No periodontitis, there is no evidence of mild, moderate, or severe periodontitis.
- Mild periodontitis, is the presence of at least 2 interproximal sites with *CAL* ≥ 3 mm and at least 2 interproximal sites with *PPD* ≥ 4 mm (not on the same tooth) or 1 site with *PPD* ≥ 5 mm.
- Moderate periodontitis, defined as at least 2 interproximal sites with *CAL* ≥ 4 mm (not on the same tooth) or at least 2 interproximal sites with *PPD* ≥ 5 mm (not on the same tooth).
- Severe periodontitis, defined as having at least 2 interproximal sites with *CAL* ≥ 6 mm (not on the same tooth) and at least 1 interproximal site with *PPD* ≥ 5 mm.

### 3.7. Self Perception

Variables that represent the oral health status and their relation to oral quality of life will be included as follows:

#### 3.7.1. Oral Health Related Quality of Life

The evaluation on Oral Health-Related Quality of Life (OHRQoL) will be carried out with the abridged version of the *Oral Health Impact Profile (OHIP-14)* that has been validated in the Mexican population (64, 65). The interview will be carried out before clinical examination. It consists of 14 questions in order to evaluate the impact of oral health conditions on people's physical, mental and social well-being. Each question has five possible answers ordered from least to most frequent (0. Never, 1. Almost never, 2. Sometimes, 3. Frequently, 4. Almost always). The instrument is scored by adding the code of each response, offering a scale from 0 to 56, where the highest score represents the greatest negative impact on quality of life.

#### 3.7.2. Diet Changes Due to Chewing Problems

Participants will be questioned as follows: *Is there any type of food that you have avoided due to problems with your teeth, dentures, or mouth in the last month?* The answer to this question will be dichotomous (Yes/No), if the answer is yes, the participants will be asked what foods they have avoided.

#### 3.7.3. Self-Perception of Need for Dental Treatment

The need for dental treatment will be assessed with the question *Do you consider that you need dental treatment?* With possible answers: No = 0, Yes = 1, Maybe = 2.

#### 3.7.4. Xerostomia

Xerostomia or dry mouth feeling will be evaluated using the *Xerostomia Inventory* that has been translated and validated into Spanish (66). The Xerostomia Inventory consists of 11 questions with five possible answers (1. Never, 2. Hardly, 3. Occasionally, 4. Sometimes, 5. Very frequently), the code of each answer is added to offer a score that goes from one to 55, where the highest score suggests the greatest severity of symptoms. Participants will be asked to select one of five possible responses for the 11 questions (see note 2 in the Supplementary Material).

### 3.8. The Functionality of a Removable Prosthesis

The functionality of a removable prosthesis, retention, stability, adaptation, and integrity of the metallic structure (in case it is present), adaptation and integrity of the acrylic base will be evaluated by following *Ettinger's criteria* (see note 3 in the Supplementary Material) (67). The presence of retainers on root surfaces will be indicated. The information will be obtained by observation from the examiner.

### 3.9. Oral Hygiene

#### 3.9.1. Sampling of Biofilm (Dental Plaque) and Calculus

The biofilm and dental calculus will be obtained from the buccal and lingual surfaces of all natural teeth present according to the criteria of *Green and Vermillion* (based on the fraction of surface free of plaque) (68). For biofilm and calculus, the percent of tooth surfaces not covered, covering up to 1/3 of the surface, between 1/3-2/3 of the surface, and >2/3 covered will be calculated (see note 4 in the Supplementary Material).

#### 3.9.2. Root Remains

To assess the consequences of untreated coronal caries the *Pulp, Ulcer, Fistula, Abscess (PUFA) Index* will be used to identify if the lesion involves the pulp, if there is the presence of ulcerations, fistulas, or dental abscesses (69). The evaluation is carried out visually without the aid of any type of instrument. A single value is assigned to each tooth (see note 5 in the Supplementary Material).

#### 3.9.3. Coronal Caries

The *DMFT index (decayed, missing, and filled teeth)* will be used to evaluate dental caries experience based on the World Health Organization caries diagnostic criteria (see note 6 in the Supplementary Material) (70).

#### 3.9.4. Number of Teeth Present

A tooth is considered to be present in the mouth when any part of its crown is visible or can be touched with the tip of the probe without displacing the soft tissue or has more than one-third of the three axial surfaces of the crown.

#### 3.9.5. Gingival Bleeding

Gingival bleeding will be measured with the *Bleeding On Marginal Probing Index (BOMP)* defined by Van der Weijden et al. (71). BOMP will be recorded after inserting a PCP UNC15 periodontal probe (WHO-Hu Friedy^®^) along the marginal gingiva (~2 mm) and held at an angle of ~60° to the longitudinal axis of the tooth according to the Angulated bleeding index (AngBI); bleeding will be scored within 30 s after probing (71).

The clinical parameters will be assessed at six sites per tooth: distobuccal, buccal, mesiobuccal, distolingual, lingual and mesiolingual. Gingival units that bleed on probing will be recorded as follows: giving non-bleeding sites a score = 0, bleeding at one point a score = 1, excessive bleeding a score = 2. This index has been used in several studies and provides specific information for every gingival unit as reported in the update by the group of Van der Weijden (72). A participant will be considered to have gingival bleeding when at least one gingival margin is rated one or higher.

## 4. Data Collection

Participants in the umbrella cohort who meet the inclusion criteria will be invited to participate in this study. The purpose of the study will be explained to them and they will be asked to sign an additional informed consent document. An appointment will be scheduled to carry out the oral health interview, dental clinical examination and sampling for the saliva and subgingival microbiota analysis at the facilities of the Graduate Studies and Research Division of SD-UNAM. The clinical examination will be performed with the person sitting in the dental chair under artificial light. It will be explained to them that strict infection control measures will be used and that all instruments will be sterile and for their exclusive use. In the event that something unusual is observed during the dental examination, the participant will be informed that it is necessary for a dentist to examine it in more detail and will be referred to the SD-UNAM clinics. To record the data an encoder will fill-in the information in the corresponding format.

**Figure 2:**
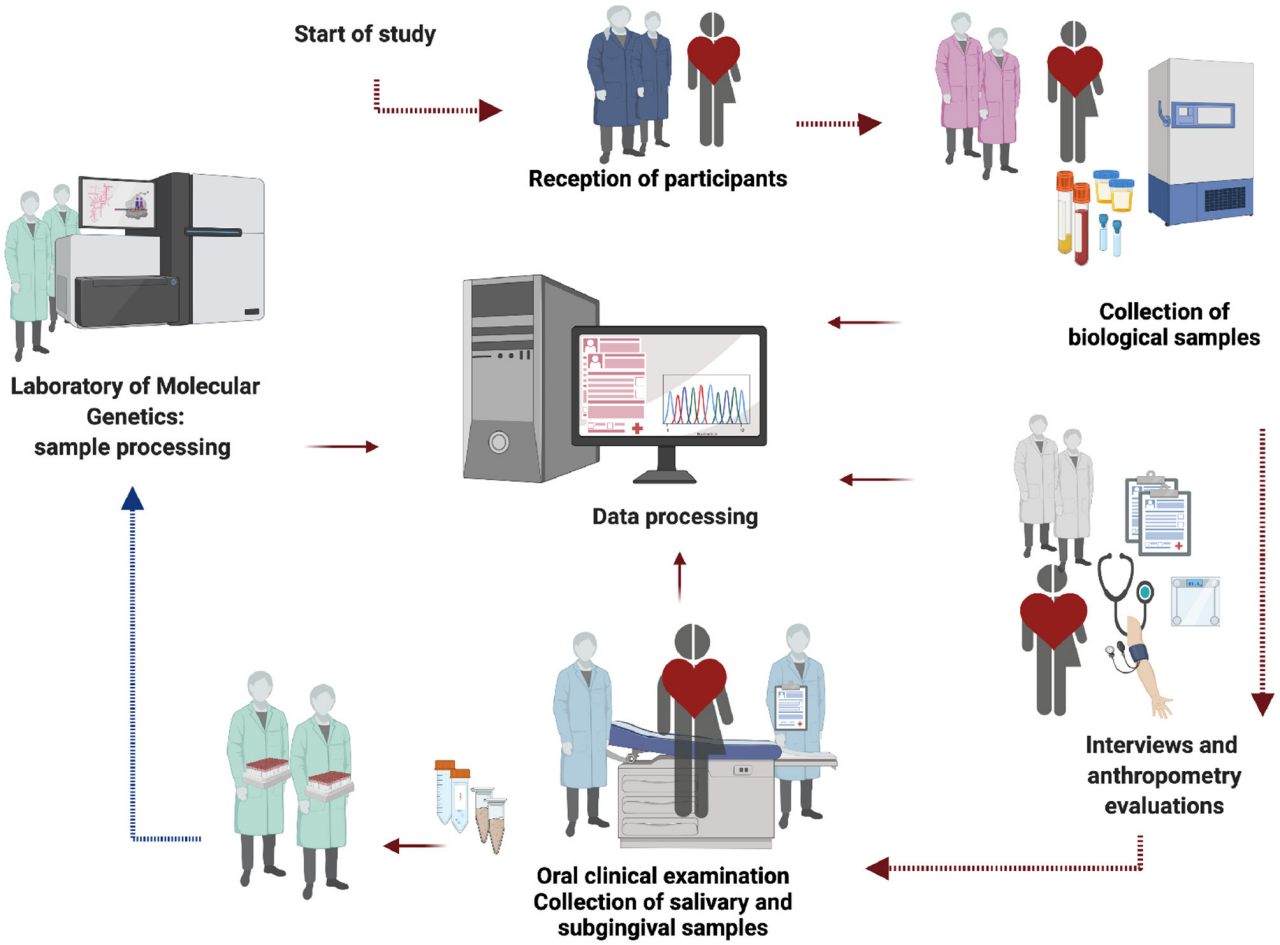
Flow chart of study. The figure represents an interview sequence, sampling procedures and data management that will be carried out in the protocol. The image generated by the authors was created with the *BioRender* App.

The full study will be organized in five exploration stages mentioned below: 1) reception of participants, 2) collection of biological samples, 3) interviews and anthropometric measurements, 4) oral examination and 5) sample processing (see Figure 2).

## 5. Oral Health Diagnosis

Participants will be evaluated every year using standardized and valid clinical measurements to identify the presence of periodontitis (see Figure 1). All participants will receive detailed information about their oral health following each evaluation and will be referred to specialized periodontal treatment whenever needed, particularly if they have severe disease. Receiving periodontal treatment, however, will depend only on each participants' decision. It should be noted that our research group will not carry out any clinical intervention or pharmacological prescription. This statement is mentioned in the informed consent for the present study.

Those participants who have started phase I periodontal therapy (scaling and root planing) and have not given it continuity (at least a couple of maintenance visits every 3 months) during the follow-up (between one evaluation and another carried out by our group) *will continue to be included in the study*. End of follow-up will be given to those participants who undergo systematic and regular periodontal treatment (at intervals of 2, 3, or 4 weeks for more than 3 months) or with forms of refractory or progressive periodontal rupture, periodontitis in a rapid rate of progression, necrotizing periodontal diseases or periodontal abscess that require regular taking of antibiotics (73–75) (see Figure 1).

### 5.1. Methods Collection

Sampling of saliva and subgingival plaque will be performed at baseline and at the end of follow-up in the participants selected for the oral microbiome analysis. All participants will be asked to stop taking antibiotics 3 months before and to stop mouthwashes—1 week before the clinical examination. History of occasional antibiotic consumption (outside sampling dates) will be recorded and analyzed. They will also be asked to stop tooth brushing 24 h before the clinical exam. They will be asked to refrain from eating, drinking anything other than water and chewing gum for 12 h prior to taking samples (76).

#### 5.1.1. Saliva Sampling

Sampling of saliva will be collected in the morning (between 7:30 and 10:30 a.m.). Fully non-stimulated saliva samples will be collected spitting saliva for at least 1 min, as previously reported (77) into a sterile and pre-labeled Falcon 15-mL tube. This process will be repeated until a total volume of 2 mL is collected. Saliva samples will be divided into two 1 mL aliquots, centrifuged for 10 min at 13,200 RPM, the supernatant will be discharged, and solutions will be re-suspended in 150 μl of TE buffer prior to DNA purification.

For the detection of all the bacterial species present in saliva and subgingival dental plaque samples, DNA samples will be previously isolated and purified for the sequencing of the conserved and hypervariable regions of the RNA of the 16S ribosomal gene (16S rRNA Seq). The gene-specific sequences used in this protocol target the 16S V3 and V4 region. They are selected from Klindworth et al. (78). This experimental strategy is an alternative that circumvents the limitations of microarray-based approaches, which by necessity have a pre-established panel of microbial species. 16S rRNA is a gene universally present in prokaryotic organisms; therefore, by using the so-called “universal primers,” it will be possible to detect all the species present in a sample. To this end we will follow the experimental design of the *Human Oral Microbiome Project*, adapting it for NGS sequencing of the 16S rRNA region (79).

#### 5.1.2. Subgingival Dental Biofilm Sampling

Following saliva collection, subgingival plaque samples will be collected for microbial evaluation analysis. After drying and isolating with cotton rolls, visible supragingival plaque will be removed and discarded. Then subgingival plaque sampling will be performed from the mesiobuccal zone of first molars (or if missing, second molars or premolars) (80, 81) of each quadrant (82, 83) with an individual sterile Gracey curette (Mini-Five #SAS11/122). The Gracey Curette will be inserted deep into the gingival sulcus of the periodontal pockets without tooth contact, angled to get in close contact to the tooth root surface to carefully scrape off the biofilm (84, 85). Then each subgingival sample will be deposited into an individual sterile tube with a buffer solution.

All samples from an individual participant will be immediately placed and dispersed into the same tube containing 150 μl of TE buffer (10 mM Tris-HCl, 0.1 mM EDTA, pH 7.6). The teeth for which the sample will be taken will be recorded in the data collection format. The tube will be placed in an IsoTherm system (IsoSafe Transport Box, 0°C and IsoRack for 1.5 mL tubes) Eppendorf^*TM*^ and delivered to the laboratory for processing within 4 h of obtaining the sample. The relative microbial abundance analysis will further proceed following the same procedure described for saliva samples in the previous subsection.

## 6. Standardization

Three examiners will be standardized. Intra-and inter-examiner reliability of 90% will be achieved for plaque, bleeding, coronal caries and root remains measurements and 70% for periodontal measurements. Examiners will also be trained to take the subgingival dental plaque and saliva samples for oral microbiome analysis.

## 7. Infection Control Practices for COVID-19

Due to the characteristics of dental settings, the risk of cross infection may be higher between dental practitioners and participants. Interpersonal transmission of Severe Acute Respiratory Syndrome Coronavirus 2 (SARS-CoV-2) the pathogen of coronavirus disease 2019 (COVID-19) occurs mainly via respiratory droplets and aerosols, as well as contact transmission (86), symptomatic patients and patients in their incubation period are also carriers of SARS-CoV-2, and it remains to be fully established whether patients in the recovering phase are a potential source of transmission (87).

The standard protective measures in daily dental clinical work are not effective enough to prevent the spread of COVID-19 (87). Good hand hygiene and disinfection of all surfaces within the dental clinic will be implemented, 70% ethanol to clean and disinfect the blood pressure cuffs or thermometers will be applied, also surface sanitizers will be included 62–71% ethanol, 0.5% hydrogen peroxide, and 0.1% (1g/L) sodium hypochlorite. After each patient's visit, surfaces will be thoroughly wiped down (88). The reusable tools and materials will be cleaned, sterilized, and carefully preserved in compliance with the Protocol for the Disinfection and Sterilization of Dental Instrument (89). Whatever objects are removed from the patient's mouth (e.g., dental prosthesis) will be also disinfected (88).

Other standardized measures of maximum caution will be followed (90–92). The personal protective equipment for the examiners will include: N-95 masks, gloves, gowns, and goggles or face shields (87). Single-use devices will be applied (e.g., BP cuffs, etc.) to avoid cross-contamination. The number of appointments for evaluation will be reduced and no companions will be permitted in the dental clinic in order to avoid unnecessary aggregation. Any staff member who has COVID-19-related symptoms or has a close family member who is confirmed with the infection is advised to undergo a medical examination and cease working immediately (87). Pre-check triages to measure and record the temperature of every staff and participants will be done as a routine procedure. Pre-check staff will previously contact the participants to ask questions about the health status, symptoms such as cough and fever, history of contact and any visit to epidemic regions within the past 14 days. A positive answer to any of these questions will postpone the date of dental sampling for at least 4 weeks.

## 8. Data Coding and Recording

The information will be recorded in a data collection instrument designed *ad hoc* for this study. All the information will be registered in three relational databases: 1) the one that contains sociodemographic information, of factors associated with the development of systemic arterial hypertension and the registry of incidence of hypertension obtained from the umbrella cohort; 2) the oral health database which will contain the information from the interview and the dental clinical examination; 3) the database that will contain the information from the computational analysis of microbiomes.

## 9. Analysis

### 9.1. Statistical Analysis

A cross-sectional and longitudinal analysis of the information will be carried out as follows:

#### 9.1.1. Cross-Sectional Analysis

Descriptive statistics (proportion, mean, standard deviation, median, mode, and range, as appropriate) will be calculated to describe the oral characteristics of the participants during each 12-month period. Univariate analyses will be performed through parametric and non-parametric tests to explore the distribution of continuous variables based on defined exposure variables. In the same way, χ^2^ tests will be performed to explore the association of categorical variables with variables of interest. Multiple analyses (logistic regression, Poisson regression, negative binomial regression, generalized linear regression, gamma regression) will be performed to explore the association of the dependent variable with one or more independent variables while controlling for covariates.

#### 9.1.2. Longitudinal Analysis

As part of the longitudinal analysis the incidence of each oral variable included in the study in 30-month periods will be evaluated. Similarly the relative risk for each oral variable included will be evaluated according to different variables considered as risk factors (alcoholism, smoking, among others) through bivariate and multivariable analyses, using generalized linear models, logistic regression models, and multinomial regression models.

Starting with the third evaluation (baseline evaluation + first follow-up + second follow-up), trajectories of oral health conditions will be identified. From these variables associated with each one will be identified (gender, marital status, occupational class, age, education, among others). In the same way trajectories will be considered as risk factors for the development of hypertension in the study participants and will be included in neural network models.

For the statistical association analysis, participants will be classified in two groups: Group 1: without periodontitis and Group 2: with periodontitis (including all those participants that according to the CDC/AAP have mild, moderate, or severe periodontitis). For the sake of this association analysis and by following the consensus of the literature (73–75), participants in Group 2 who start periodontal treatment but do not continue with it until remission will be kept in this group and considered with periodontitis. However, a note will be made with details on the type, the extent of their treatment and the effects on their periodontal health, derived from their periodical periodontal evaluation.

This information will be used for a further *a posteriori* analysis. The information about the extent of their periodontal disease (mild, moderate and severe) will be also considered a posteriori. It is worth noticing that *a posteriori cluster analysis*, for instance, Propensity Score evaluation (93, 94) is not unusual in prospective observational studies subject to constraints, as is this case (95–99).

### 9.2. Microbiota Analysis

Bacterial DNA isolation and purification will be performed using the Qiagen Gentra Puregene Yeast/bacteria Kit, following the manufacturer's instructions to isolate DNA from Gram positive bacteria.

#### 9.2.1. Amplification of the 16S rRNA Gene

The purified DNA samples will be amplified with universal F24/Y36 primers to build wide coverage libraries. Additional libraries will be generated to expand the coverage of *Bacteroidetes* from the TM7/SR1 and *Spirochetes*/*Synergistetes* groups that will be amplified with primers of type F24/F01 or F24/M98. PCR cycles will be performed in thin-walled tubes in a Perkin Elmer 9700 (or higher) thermal cycler. The reaction mixture (50 μl final volume) will contain 1 μl of purified DNA template, 20 pmol of each primer, 40 nmol dNTPs, 2.5 units of Taq polymerase Platinum (Invitrogen, Carlsbad, CA) and 5 μl buffer 10X for PCR (200mM Tris-HCL, pH 8.4, 500 mM KCl). Following these steps, the warm-start protocol of tempered-elongation amplification will be followed and optimal amplicon sizes will be determined using agarose gels, following the standard procedure with the Qiagen gel extraction kit (Qiagen, Valencia, CA).

#### 9.2.2. Library Screening and 16S Sequencing

These procedures will be performed using 16S easy kits following the procedures for sequencing in Illumina technology (San Diego, CA).

#### 9.2.3. Design of the Computational Analysis of Microbiomes

The computational analysis of the microbiota data, and the strategy of curing, documentation and archiving of the metagenomic data will be carried out according to the *CAMI* guidelines (Critical Assessment of Metagenome Interpretation) that emphasizes the implementation of workflows that ensure reproducibility, following the Docker packaging approach in “Bioboxes” adapted for the analysis of the 16S rRNA region (100, 101).

#### 9.2.4. Design of the Association Studies of the Microbiome With Clinical, Social Variables, and Risk Factors

In order to determine the relationships between the oral subgingival microbiome with response variables (clinical, biochemical, socioenvironmental, lifestyle, among other risk factors) a hybrid design has been chosen that combines classic approaches to epidemiology and biomedical statistics (proportional hazards models, Cox regressions, multivariate and logistic, transformed variables, etc.) with contemporary analysis methodologies based on computational learning ideas (neural networks, deep learning, support vector machines, Bayes naive-type models, decision trees, etc.). The choice between models will consider principles of maximum likelihood and maximum parsimony.

## 10. Design and Implementation of the Data Management Plan

The purpose of the Data Management Plan is to establish guidelines on how the data will be treated during the course of the project and what will happen to this data at the end of the project. This document will detail what will be done with the data from its collection, its organization, pre-processing, analysis, quality controls, data preservation techniques, and documentation of the databases. Likewise, the conditions of use, dissemination, sharing options, embargoes and limitations are stipulated. To this end we will follow the general recommendations of the *Clinical Data Management Plan Committee* (102).

## 11. Design of the Communication Strategy and Socialization of the Results

Given that HBP is an important public health problem, that its pharmacological treatment is complex and expensive, and that its consequences (individually and collectively) are serious, we consider a patient-centered approach to primary and secondary prevention, as the strategy with the greatest potential to promote the results of this study. For this reason it is especially relevant in the Mexican context in which this project is immersed to have an agile strategy for communicating the results and scope of our work, as well as for the socialization of knowledge. We believe that this will allow to have a direct positive impact on the population, particularly in participants at risk who should be well informed about the association between HBP and periodontitis, to have access to timely counseling to identify risk factors, and to receive timely medical and dental attention (103, 104).

Additionally, our results may have an impact on public policy through recommendations for direct government actions and programs, whose implementation is, by their very nature, of much longer scope. Especially in view of the next months or years, while the COVID19 pandemic remains, and time after, we will have to implement novel strategies in public dental health and a multilevel approach for hypertension control (8, 105). For example, by using teledentistry and telemedicine, mobile health applications or the use of social networks as contact means to follow and communicate with patients (106–109).

## 12. Discussion

Cardiovascular diseases and arterial hypertension are complex patho-phenotypes responsible for a large burden of morbidity and mortality worldwide. Early diagnosis and interventions are key to better control these diseases. Hence, the current protocol study aims to understand the relationship between the onset of arterial hypertension, periodontitis and the role that periodontal microbiome, as well as social determinants and risk factors that may be playing in the interrelationship between these issues.

The present protocol has been designed as a sub-cohort analysis of the larger cohort investigating the factors behind the onset of hypertension (35). This sub-cohort analysis has been planned to include a close follow-up of participants for which studies on blood pressure, periodontal health, and the oral and subgingival microbiome, will be supplemented with information on participants' lifestyle and nutritional habits, social determinants of health and risk factors.

These studies will be carried out by a multidisciplinary, multi-centric team that incorporates researchers from university settings (represented by the Graduate Division of the School of Dentistry at the National Autonomous University of Mexico), federal government institutions represented by three of Mexico's National Institutes of Health (National Institute of Cardiology Ignacio Chávez, National Institute of Geriatrics and National Institute of Genomic Medicine), and the support of researchers from the National Council on Science and Technology. As stated, the team will apply the best practices in dental public health and epidemiology, supported by rigorous sampling and laboratory protocols, state of the art statistical and computational analytic methods and a carefully designed and executed data management and data policy plan. All procedures will be performed following strict safety considerations; both in the management and sampling of participants, and in the processing of biological samples (even more stringent in the face of the recent COVID-19 pandemic).

This project has been planned with strong ethical foundations and respect to the participants' confidential or sensitive information. In view of the importance of translating basic findings into actionable interventions, this protocol will consider the implications of our findings, both in the generation of public policy, and for population empowerment, via the establishment of primary prevention—highlighting the relationship between oral and cardiovascular health. This latter set of interventions will be supported by a carefully planned science communication and health promotion strategy. All of the previously discussed goals are in line with our general view to contribute to the development of early interventions aimed at diminishing the devastating consequences of cardiovascular diseases, in particular by highlighting the relationship between periodontitis and arterial hypertension and the mediation of the oral subgingival microbiome.

### 12.1. Limitations of the Study

It should be noticed that any project involving research in human populations is subject to limitations that need to be acknowledged and properly contextualized in the discussion of the results and scope of the project. In the present case the following constraints are to be considered:

- This study will be carried out in an urban population from a large metropolitan area, composed of relatively young and middle-aged adults, in general health and health-aware enough to voluntarily enroll in this study. These population features may indeed represent a potential selection-bias that must be considered.
- Other variables that have been argued to contribute to the associations of oral microbiota with hypertension via periodontitis, but are not measured in this study, such as markers of endothelial function and systemic presence of pathogens, will not be considered in the statistical models since we are not measuring them directly. Indirect evidence of these, if available, will be included in the results' discussion and perspectives.
- Along the previous lines, we should also note, that aside from the effects of the oral periodontal microbiome, there is a likely involvement of the (more abundant) gut microbiota, that can be a confounding factor for parameters such as serum energetic metabolites, blood pH, endothelial function and others. Concurrent measurement of the gut microbiota is, however, out of the scope of the present study.

## Ethics Statement

This study has been registered and approved by the Research and Ethics Committee of the School of Dentistry, Universidad Nacional Autónoma de México (CIE/0308/05/2019) and the National Institute of Genomic Medicine (CEI/2020/12). The umbrella cohort was approved by the Institutional Bioethics Committee of the National Institute of Cardiology-Ignacio Chavez (INC-ICh) under code 13-802. The patients/participants provided their written informed consent to participate in this study.

## Author Contributions

MM-G, RC-P, MV, SB-Y, and EH-L conceived the project. MM-G, RC-P, AR-H, MV, SB-Y, and EH-L planned the methodology and analyzes and wrote the original draft preparation. RC-P, SS-M, and EH-L designed and developed the computational strategy. MV, SB-Y, and EH-L acquired the funds. MM-G, AR-H, RC-P, SB-Y, and EH-L wrote and reviewed the manuscript. All authors read and approved the final version of the protocol manuscript.

## Funding

This research was funded by the DGAPA-UNAM PAPIIT IN222820 research grant. As well as by Cátedras CONACYT project and intramural funds from the National Institute of Genomic Medicine. The funders had no role in study design, data collection and analysis, decision to publish, or preparation of the manuscript.

## Conflict of Interest

The authors declare that the research was conducted in the absence of any commercial or financial relationships that could be construed as a potential conflict of interest.

## Publisher's Note

All claims expressed in this article are solely those of the authors and do not necessarily represent those of their affiliated organizations, or those of the publisher, the editors and the reviewers. Any product that may be evaluated in this article, or claim that may be made by its manufacturer, is not guaranteed or endorsed by the publisher.
