# Supplementary material for: Incidence of Arterial Hypertension in People With Periodontitis and Characterization of the Oral and Subgingival Microbiome: A Study Protocol
Source: Front Cardiovasc Med. 2022 Jan 7;8:763293. doi: 10.3389/fcvm.2021.763293 (PMC8776993; doi:10.3389/fcvm.2021.763293)
Supplement: Supplementary file 1 [file Data_Sheet_1.PDF]

Supplementary Material

DESCRIPTION OF THE PROSPECTIVE LONGITUDINAL STUDY TLALPAN 2020 COHORT

The Tlalpan 2020 cohort is an observational, longitudinal, prospective study that is conducted in Mexico City, Mexico at the *Instituto Nacional de Cardiología Ignacio Chávez* (INC-ICh), one of Mexico's National Institutes of Health and a public flagship hospital institution for the treatment of cardiovascular diseases in Mexico (Cruz-Ávila et al., 2020). The Tlalpan 2020 study was approved by the Research Ethics Board for Biomedical Research in Humans of INC-ICh under number 13-802 (Colín-Ramírez et al., 2017). The enrollment started in September 2014 to September 2019.

Healthy volunteers are women and men residents of Mexico City, between 20 and 50 years old. At the beginning of recruitment no participants used drugs to treat hypertension, since none of them suffer hypertension. Individuals previously diagnosed with some disease such as diabetes mellitus, dysthyroidism, cerebrovascular disease, ischaemic cardiomyopathy, acute coronary syndrome, cancer with an effect on survival, pregnant women and those taking medications that have an effect on blood pressure, also persons with cognitive and mental disabilities are excluded. All volunteers gave written informed consent to participate in Tlalpan 2020 study.

The participants are evaluated every 2 years over a period of 10 years or until they develop hypertension (primary outcome variable). During the initial visit (baseline), clinical, anthropometric, biochemical, diet, physical activity, stress, sleep quality, sociodemographic data, personal and family pathological antecedents, and alcohol and tobacco consumption are collected. All instruments are applied by personnel trained at INC-ICh.

Evaluations and instrumentation: Tlalpan 2020 cohort

Anthropometry and clinical parameters

High blood pressure (HBP) for the Tlalpan 2020 cohort was defined as a systolic blood pressure $\geq$ 140 mm Hg and/or a diastolic blood pressure $\geq$ 90 mm Hg (Colín-Ramírez et al., 2017). The present study uses an updated, more precise and broader scope definition of hypertension, including office and home blood pressure measurements (see main text). Evaluation of anthropometric measurements (weight, height and waist circumference) are performed with the patient fasting, shoeless and wearing a hospital gown in accordance with the procedures described by 'The International Society for the Advancement of Kinanthropometry' (ISAK). A mechanical column scale (SECA 700) with a capacity of 220 kg and precision of 0.05 kg is used and the weight is recorded to the nearest 100 g. Height will be measured with a stadiometer SECA 220. Waist circumference is measured at the level of the narrowest point between the lower costal border and the iliac crest by using a measuring tape made of glass fibre BodyFlex, with a length of 150 cm and precision of 1mm. Body Mass Index (BMI) is categorized based on World Health Organization definitions (normal, overweight, or obesity) (World Health Organization, 2000).

Biological test

The biochemical samples are measured in automatic analyzers at the Central Laboratory of INC-ICh. Blood samples are obtained after an overnight fast of 12 hours: Fasting plasma glucose (70-105 mg/dl), triglycerides (40-200 mg/dl), low density lipoprotein cholesterol (LDL-C) (80-130 mg/dl), high-density

lipoprotein cholesterol (HDL-C) (women: > 50 mg/dl and men: > 40 mg/dl), total cholesterol (140-200 mg/dl), uric acid (women: 3.80-6.20 mg/dl and men: 4.80-8.00 mg/dl), serum creatinine (women: 0.60-1.00 mg/dl and men: 0.70-1.30 mg/dl), atherogenic Index (LDL/HDL, elevated defined as a value > 4) and serum sodium, (136.00-145.00 mmol/l). Altered lipid profile is defined according to the Adult Treatment Panel III criteria: high total cholesterol when >5.2mmol/L, low HDL-C when <1.0mmol/L for men and <1.3mmol/L for women, high LDL-C when >3.4mmol/L and high triglycerides when 1.7mmol/L.

Urine sample data in 24 hours (24 h) are also obtained. For a correct urine collection, the participant is given precise and clear indications, several days in advance of their appointment (discard the first urine in the morning and collect all urine for a period of 24 hours, including the first urine of the following morning, which will be the day of the appointment).

Urinary sodium and potassium are determined by the ion selective electrode method, and urinary creatinine is determined by Jaffe's colorimetric assay using an automated analyser. The urine sample is considered complete when urinary creatinine levels are within the standard creatinine excretion rate (133–221 μmol/ kg/24hours for men and 88–177 μmol/kg/24hours for women) (Wielgosz et al., 2016). The reference values of urinary variables are following: for creatinine in women between 740-1570 mg/24 h and for men between 1040-2350 mg/24 h, for sodium between 40.00-220.00 mmol/24 h and for potassium excretion between 25.00-125.00 mmol/24 h. Sodium and potassium excretion is reported in mmol/24 h (or equivalently mEq/24 h).

Nutritional and lifestyle habits

Also, antecedents of alcohol and tobacco frequency of consumption are collected (daily, every other day, every weekend, every 2 weeks, once a month or less than once a month). Participants who report smoking at least 100 cigarettes in their lifetime and who, at the time of the survey, smoked either every day or some days would be classified as current smokers (Malarcher et al., 2009). The physical activity antecedent (as measured by the long version of *International Physical Activity Questionnaire, IPAQ: categorized into low, moderate, or high physical activity levels* (Craig et al., 2003)) and psychological stress level (as determined by the *State-Trait Anxiety Inventory, STAI* Spanish version; categorized into low, moderate, or severe psychological stress (Spielberger, 2013)) are obtained. Also, sleep disorders are evaluated by the Spanish-language Medical Outcomes Study-Sleep scale of 12 items to assess the last week of sleep. Dietary intake is evaluated using a semiquantitative questionnaire validated in a Mexican population developed by the National Institute of Public Health of Mexico (Hernández-Avila et al., 1998).

Social determinants of health

Through a personal interview, the following demographic and socioeconomic data are obtained: Marital status (single, married and other), educational level concluded (elementary school, junior high school, higher and postgraduate), occupational class (student, business executive, housekeeper, professional, manually qualifies, manually unqualified, other and unemployed). Also, social development index strata is calculated from the housing, geographic location within the 16 boroughs of Mexico City self-reported by the participants (Martínez-García et al., 2021).

ORAL CLINICAL EXAMINATION

The projected sample size for periodontitis and incidence of hypertension will be 1212 of those participating in the Tlalpan 2020 cohort.

Note 1. Periodontitis

Six sites in all teeth (distobuccal (DB), buccal (B), mesiobuccal (MB), distolingual (DL), lingual (L) and mesiolingual (ML)) will be measured using a PCP UNC15 periodontal probe (Holtfreter et al., 2015). Periodontal attachment loss will be assessed calculating the distance from the free gingival margin (FGM) to the cemento-enamel junction (CEJ), and the distance from the FGM to the bottom of the pocket. Where the gingival margin has receded and the CEJ is exposed, the distance from the CEJ to the gingival margin will be assigned a negative value.

Note 2. Xerostomia

Xerostomia or dry mouth feeling will be evaluated using the Xerostomia Inventory that has been translated and validated into Spanish (Serrano et al., 2016). The xerostomia inventory consists of the following 11 items:

1. I sip liquids to aid in swallowing food.
2. My mouth feels dry when eating a meal.
3. My lips feel dry.
4. I have difficulties swallowing certain foods.
5. My mouth feels dry.
6. I get up at night to drink.
7. I have difficulty in eating dry foods.
8. My eyes feel dry.
9. I suck sweets or cough lollies to relieve dry mouth.
10. The inside of my nose feels dry.
11. The skin of my face feels dry.

The participants will be asked to indicate which one of five response options best described their symptoms over the preceding 2 weeks. The response options can be:

- (Scoring 1) Never.
- (Scoring 2) Hardly ever.
- (Scoring 3) Occasionally.
- (Scoring 4) Fairly often.
- (Scoring 5) Very often.

Note 3. The Functionality of a removable prosthesis

The functionality of dental prostheses will be evaluated by following Ettinger's criteria (Ettinger and Jakobsen, 1997):

Stability. One finger will be placed on the premolar region on each side, then will be attempt to rock the denture from side to side, and the score will be as follows:

- (Scoring 0) No movement.
- (Scoring 1) Some movement but functionally adequate.

- (Scoring 2) Movement/treatment required.

Retention. The fingers will be placed at the incisor region on the lingual or palatal surface, then will be attempt to push the denture labially, and the score will be as follows:

- (Scoring 0) Good seal.
- (Scoring 1) Some movement but functionally adequate.
- (Scoring 2) Movement/treatment required.

Extension. A visual inspection will be made of the dentures in and out of the mouth, and the score will be as follows:

- (Scoring 0) Covers anatomical landmarks; no cracks or under– or over-extension.
- (Scoring 1) Overextension/easily correctable.
- (Scoring 2) Underextension, cracks or holes or a broken denture whieh needs treatment.

Occlusion. A visual inspection will be made in occlusal zone of the dentures, and the score will be as follows:

- (Scoring 1) Maximum intercuspation without significant wear.
- (Scoring 2) Occlusal wear or loss of contact requiring treatment.

Note 4. Oral Hygiene Index

The evaluation will only be performed on fully erupted teeth. Third molars will be excluded from the evaluation. The oral hygiene examination will be conducted according to the criteria of Green and Vermillion (Greene and Vermillion, 1960), in the following way:

- First, the buccal and, second, the lingual surfaces of the teeth in the upper right posterior segment will be inspected and will be scored for dental plaque.
- Then the labial and lingual surfaces of the upper anterior teeth will be classified.
- Finally, the buccal and lingual surfaces of the teeth in the upper left posterior segment will be examined and will be scored.
- The lower arch inspection proceeds will be in the same manner, but from left to right.
- This routine will be repeated in the inspection for calculus after the dental plaque recordings have been completed.

Dental calculus will be differentiated by location on the tooth in relation to the free gingival margin:

- *Supragingival calculus* will be indicated by deposits located occlusal to the free gingival margin and usually white to yellowish brown in color.
- *Subgingival calculus* will be indicated by deposits located apically to the free gingival margin, which are usually light brown to black in color because of inclusion of blood pigments.

The scores and criteria for dental plaque will be:

- (Scoring 0) No dental plaque or stain present.
- (Scoring 1) Soft dental plaque covering not more than 1/3 of the tooth surface, or the presence of extrinsic stains without other dental plaque regardless of surface area covered.

- (Scoring 2) Soft dental plaque covering more than 1/3, but not more than 2/3, of the exposed tooth surface.
- (Scoring 3) Soft dental plaque covering more than 2/3 of the exposed tooth surface.

The scores and criteria for calculus will be:

- (Scoring 0) No calculus present.
- (Scoring 1) Supragingival calculus covering not more than 1/3 of the exposed tooth surface.
- (Scoring 2) Supragingival calculus covering more than 1/3 but not more than 2/3 of the exposed tooth surface or the presence of individual flecks of subgingival calculus around the cervical portion of the tooth or both.
- (Scoring 3) Supragingival calculus covering more than 2/3 of the exposed tooth surface or a continuous heavy band of subgingival calculus around the cervical portion of the tooth or both.

Note 5. PUFA index

PUFA index will be used to assess the presence of oral conditions resulting from untreated caries (the presence of either a visible pulp, ulceration of the oral mucosa due to root fragments, a fistula or an abscess). Lesions in the surrounding tissues that are not related to a tooth with visible pulpal involvement as a result of caries will be not recorded. The assessment will be carried out visually without the use of an instrument. Only one score will be assigned per tooth (in capital letters for permanent dentition). The PUFA score will be calculated in the cumulative way and will represent the number of teeth that meet the PUFA diagnostic criteria (Monse et al., 2010).

The codes and criteria for the PUFA index will be the following:

- **P:** Pulpal involvement will be recorded when the opening of the pulp chamber is visible or when the coronal tooth structures have been destroyed by the carious process and only roots or root fragments remain. No probing will be performed to diagnose pulpal involvement.
- **U:** Ulceration due to trauma from sharp pieces of tooth will be recorded when sharp edges of a dislocated tooth with pulpal involvement or root fragments have caused traumatic ulceration of the surrounding soft tissues (tongue or buccal mucosa).
- **F:** Fistula will be registered when a pus releasing sinus tract related to a tooth with pulpal involvement is present.
- **A:** Abscess will be scored when a pus containing swelling related to a tooth with pulpal involvement is present.

Note 6. DMFT index

The DMFT index (decayed, missing, and filled teeth) will be used to evaluate the experience of dental caries status based on the World Health Organization caries diagnostic criteria (World Health Organization, 2013). The codes and criteria for the DMFT index will be the following:

- (Scoring 0) Sound: A crown/root will be coded as sound if it shows no evidence of treated or untreated clinical caries. The stages of caries that precede cavitation, as well as other conditions similar to the early stages of caries, will be excluded because they cannot be reliably identified in most field conditions in which epidemiological surveys are conducted.

- (Scoring 1) Carious crown: Caries will be recorded as present when a lesion in a pit or fissure, or on a smooth tooth surface, has an unmistakable cavity, undermined enamel, or a detectably softened floor or wall. Carious root - Caries will be recorded as present when a lesion feels soft or leathery on probing with the CPI probe.
- (Scoring 2) Filled crown, with caries: A crown will be considered filled, with caries, when it has one or more permanent restorations and one or more areas that are decayed. Filled root, with caries - A root will be considered filled, with caries, when it has one or more permanent restorations and one or more areas that are decayed.
- (Scoring 3) Filled crown, with no caries: A crown will be considered filled, without caries, when one or more permanent restorations are present and there are no caries anywhere on the crown. Filled root, with no caries - A root will be considered filled, without caries, when one or more permanent restorations are present and there are no caries anywhere on the root.
- (Scoring 4) Missing tooth, due to caries: This code will be used for permanent teeth that have been extracted because of caries and are recorded under coronal status.
- (Scoring 5) Permanent tooth missing due to any other reason: This code will be used for permanent teeth deemed to be absent congenitally, or extracted for orthodontic reasons or because of periodontal disease, trauma, etc.
- (Scoring 6) Fissure sealant: This code will be used for teeth in which a fissure sealant has been placed on the occlusal surface or, in a pit or for teeth in which the occlusal fissure has been enlarged with a rounded or flame-shaped bur, and a composite material has been placed.
- (Scoring 7) Fixed dental prosthesis abutment, special crown or veneer: This code will be used under coronal status to indicate that the tooth forms part of a fixed bridge abutment. This code can also will be used for crowns placed for reasons other than caries and for veneers or laminates covering the labial surface of a tooth, on which there is no evidence of caries or a restoration.
- (Scoring 8) Unerupted tooth (crown): This classification will be used only for a tooth space with an unerupted permanent tooth.
- (Scoring 9) Not recorded.

REFERENCES

Cruz-Ávila HA, Vallejo M, Martínez-García M, Hernández-Lemus E. Comorbidity networks in cardiovascular diseases. *Front Physioly* **11** (2020) 1009. doi:10.3389/fphys.2020.01009.

Colín-Ramírez E, Rivera-Mancía S, Infante-Vázquez O, Cartas-Rosado R, Vargas-Barrón J, Madero M, et al. Protocol for a prospective longitudinal study of risk factors for hypertension incidence in a mexico city population: the tlalpan 2020 cohort. *BMJ Open* **7** (2017) e016773. doi:10.1136/bmjopen-2017-016773.

World Health Organization. *Obesity: preventing and managing the global epidemic*. 894 (World Health Organization) (2000).

Wielgosz A, Robinson C, Mao Y, Jiang Y, Campbell NR, Muthuri S, et al. The impact of using different methods to assess completeness of 24-hour urine collection on estimating dietary sodium. *J Clin Hypertens* **18** (2016) 581–584. doi:10.1111/jch.12716.

Malarcher A, Shah N, Tynan M, Maurice E, Rock V, et al. State-specific secondhand smoke exposure and current cigarette smoking among adults-united states, 2008. *MMWR Morb Mortal Wkly Rep* **58** (2009) 1232–1235.

Craig CL, Marshall AL, Sjöström M, Bauman AE, Booth ML, Ainsworth BE, et al. International physical activity questionnaire: 12-country reliability and validity. *Med Sci Sports Exerc* **35** (2003) 1381–1395. doi:10.1249/01.MSS.0000078924.61453.FB.

Spielberger CD. *Anxiety: Current trends in theory and research* (Elsevier) (2013).

Hernández-Avila M, Romieu I, Parra S, Hernández-Avila J, Madrigal H, Willett W. Validity and reproducibility of a food frequency questionnaire to assess dietary intake of women living in mexico city. *Salud Publica Mex* **40** (1998) 133–140. doi:10.1590/s0036-36341998000200005.

Martínez-García M, Gutierrez-Esparza GO, Roblero-Godinez JC, Marin-Perez DV, Montes-Ruiz CL, Vallejo M, et al. Cardiovascular risk factors and social development index. *Front Cardiovasc Med* **8** (2021) 68. doi:10.3389/fcvm.2021.631747.

Holtfreter B, Albandar JM, Dietrich T, Dye BA, Eaton KA, Eke PI, et al. Standards for reporting chronic periodontitis prevalence and severity in epidemiologic studies: Proposed standards from the joint eu/usa periodontal epidemiology working group. *Journal of clinical periodontology* **42** (2015) 407–412. doi:10.1111/jcpe.12392.

Serrano C, Fariña MP, Pérez C, Fernández M, Forman K, Carrasco M. Translation and validation of a spanish version of the xerostomia inventory. *Gerodontology* **33** (2016) 506–512. doi:10.1111/ger.12196.

Ettinger RL, Jakobsen JR. A comparison of patient satisfaction and dentist evaluation of overdenture therapy. *Community Dent Oral Epidemiol* **25** (1997) 223–227. doi:10.1111/j.1600-0528.1997.tb00930.x.

Greene JC, Vermillion JR. The oral hygiene index: a method for classifying oral hygiene status. *J Am Dent Assoc* **61** (1960) 172–179.

Monse B, Heinrich-Weltzien R, Benzian H, Holmgren C, van Palenstein Helderman W. Pufa–an index of clinical consequences of untreated dental caries. *Community Dent Oral Epidemiol* **38** (2010) 77–82. doi:10.1111/j.1600-0528.2009.00514.x.

World Health Organization. *Oral health surveys: basic methods* (World Health Organization) (2013).
